# Effect of sodium valproate on the sleep structures of epileptic patients

**DOI:** 10.3892/etm.2014.1593

**Published:** 2014-02-28

**Authors:** HUI ZHANG, YUECHUN LI, XIUE LI, GUORONG LIU, BAOJUN WANG, CHUNHUA LI

**Affiliations:** Department of Neurology, Baotou Central Hospital, Baotou, Inner Mongolia Autonomous Region 014040, P.R. China

**Keywords:** sodium valproate, polysomnography, sleep structures, bodyweight, epilepsy

## Abstract

The aims of the present study were to investigate the effect of sodium valproate (VPA) on the sleep structures of epileptic patients and the correlation of these effects with patient weight gain. A total of 60 epileptic patients were divided into three groups: E-AED I (VPA administration for a duration of <3 months), E-AED II (VPA administration for a duration of >3 months) and ECO (without VPA) groups, for polysomnography monitoring. When the E-AED II group was compared with the E-AED I and ECO groups, non-rapid eye movement sleep phase 1 was significantly prolonged (92.10±48.24, 29.50±10.61 and 23.94±13.27 min, respectively; P<0.01), rapid eye movement sleep was significantly shortened (70.82±17.69, 116.99±12.90 and 126.19±35.01 min, respectively; P<0.01), sleep efficiency was significantly reduced (89.39±2.55, 91.98±2.53 and 91.96±3.14%, respectively; P<0.01), the number of times of that the patients awoke was significantly increased (7.25±2.86, 2.55±1.42 and 2.40±1.39, respectively; P<0.01) and the number of REM phases throughout the night was significantly reduced (P<0.01). There were no significant differences for the various sleep parameters between the E-AED I and ECO groups. Therefore, VPA is capable of inducing sleep structure disorders in epileptic patients. In addition, these disorders begin 3 months following the administration of VPA, which indicates that these disorders may be associated with VPA-induced weight gain.

## Introduction

Epilepsy is a chronic brain disease induced by various etiologies with an incidence rate of 3–7%. The disease is characterized by sudden, transient and repeated central nervous system dysfunctions caused by the abnormal discharge of brain neurons. To date, the majority of treatments for epilepsy depend on drug control. Studies on epilepsy have observed that epileptic patients experience sleep problems, with a specific study attributing this to three aspects, namely epileptiform discharge, antiepileptic drug application and inherent sleep problems of epileptic patients (1). Among these factors, the influence of antiepileptic drugs on the sleep structures of epileptic patients should not be ignored, as correcting the dyssomnia of epileptic patients may contribute to their treatment.

Sodium valproate (VPA) is a broad spectrum antiepileptic drug that is widely used in clinical practice due to its high degree of efficacy and minimal sides effects. VPA is the preferred drug for the control of absence, myoclonic and generalized tonic-clonic seizures. Furthermore, VPA has a high degree of efficacy for other complex and refractory epilepsies.

Previous studies have investigated the effect of VPA on the sleep structures of epileptic patients, but the results of the studies vary. Roder and Wolf (2) observed that in comprehensive epilepsy patients, VPA was capable of increasing non-rapid eye movement (NREM) sleep phase 1 and reducing NREM sleep phase 2, but did not alter slow wave sleep (SWS), rapid eye movement (REM) sleep, sleep latency or the number of times that the patient awoke in the night. In patients presenting with absence seizures, VPA administration made it difficult for epileptic patients to fall asleep and also induced daytime drowsiness (2). Legros and Bazil (3) observed that VPA damaged the sleep structures of epileptic patients by increasing NREM sleep phase 1 and reducing REM sleep, thus, causing sleep deficiency and daytime drowsiness (3). Sammaritano and Sherwin (4) observed that VPA did not have a marked influence on sleep and only slightly increased NREM sleep phases 3 and 4, while VPA had no effect on the percentage of REM sleep or total sleep time (4). In certain studies, VPA was shown to stabilize sleep and cause sleep structures to become more regular (5). Furthermore, other studies have indicated that epileptic patients who had been administered VPA presented with sleep problems, including daytime drowsiness, fatigue, difficulty concentrating, seizure worsening and circadian rhythm disorder (3). It is possible to enhance epileptic seizure control and reduce medication by avoiding sleep disruption.

In recent years, of the side effects caused by VPA, weight gain has received considerable attention and has been shown to be group one of the reasons for the reduced compliance of VPA for epileptic patients. Luef *et al* (6) identified that 20–70% of patients receiving VPA as an antiepileptic treatment presented with weight gain. Demir and Aysun (7) observed that the incidence of obesity in epileptic patients being administered VPA ranged between 37.5 and 49%. Weight gain is associated with sleep apnea and ~13% of epileptic patients present with moderate to severe sleep-disordered breathing, particularly obstructive sleep apnea (8). For epileptic patients, the diagnosis and treatment of sleep apnea may improve the seizures (9).

Therefore, the present study investigated the effects of VPA on the sleep structures of epileptic patients and the correlation of these effects with the duration of VPA administration and VPA-induced weight gain. In the current study, polysomnography monitoring was performed throughout the night for 20 patients with epilepsy who had been administered VPA for <3 months (E-AED I group), 20 patients with epilepsy who had been administered VPA for >3 months (E-AED II group) and 20 patients with epilepsy who were not administered VPA (ECO group). The data were monitored and compared in order to investigate the correlation between the changes in the sleep structures of epileptic patients receiving VPA with the duration of VPA administration and the VPA-induced weight gain of epileptic patients.

## Subjects and methods

### Subjects

A total of 60 epileptic patients treated in the Neurology Department of Baotou Central Hospital (Baotou, China) participated in this study. All patients were diagnosed with epilepsy by the detailed enquiry of disease history, physical examination, long-term dynamic electroencephalogram and other auxiliary examinations (including MRI of head, flair of hippocampus, blood electrolytes and blood routine examination). Each patient in the test groups were administered VPA only and achieved the specified drug concentration in the blood (effective drug concentration range in the blood, 50–100 μg/ml). For patients receiving VPA, 20 patients had a treatment duration of <3 months and were classified as test group I (E-AED I) and 20 patients had a treatment duration of >3 months and were classified as test group II (E-AED II). There were an additional 20 cases of diagnosed epileptic patients who did not receive VPA. These patients were not receiving VPA at the time, but had been administered VPA previously (the drug had been completely metabolized) and were classified as the control group (ECO). In the E-AED I group, there were 9 males and 11 females, with ages ranging between 4 and 24 years. In the E-AED II group, there were 8 males and 12 females, with an age range between 5 and 24 years. In the ECO group, there were 9 males and 11 females, with ages ranging between 4 and 35 years. This study was conducted in accordance with the Declaration of Helsinki and with approval from the Ethics Committee of Baotou Central Hospital. Written informed consent was obtained from all the participants.

### Polysomnography monitoring

For the 60 patients with epilepsy, polysomnography data for whole-night natural sleep were acquired using Sandman Elite polysomnography (Siemens Healthcare Diagnostics Inc., Crumlin, UK) in the sleep monitoring room at the Neurology Department of Baotou Central Hospital. The specific schedule was dependent upon each participant’s sleeping habits. The time at which the lights were turned off was taken to be the start point for the recording and the on-bed period was taken as the recording range (this did not include any time taken for urination). The vigil awaking time following the last REM phase was taken to be the end point for the recording.

Once the sleep monitoring was complete, Sandman Elite sleep analysis software was used to conduct an automatic analysis of the sleep results of the patients and subsequent manual correction was performed. Initially, sleep was staged at one frame/30 sec, according to the Thimgan (10) and Kales (11) standards, and subsequently all the sleep indicators were analyzed.

The emphasis of this study was to stage the whole-night sleep of subjects and analyze their sleep structures. Human brain activity is divided into three states: Waking state, NREM sleep state and REM sleep state. NREM sleep may be further divided into phases 1–4. Phases 3 and 4 of NREM sleep may be collectively known as SWS or δ sleep. To date, the standard summarized by Rechtschaffen and Kales in 1968 is used for sleep staging in children >6 months-old and adults. The basic principle of sleep staging is to artificially divide the physiological record of the whole-night sleep into hundreds of records of one frame/30 sec and perform staging frame by frame.

### Statistical analysis

Measurement data were expressed as the mean ± SD. The normal distribution test was initially performed for various groups (Kolmogorov–Smirnov test) and one-way analysis of variance was used for comparisons between three groups. In the instance of non-normal distribution, a non-parametric test was used to evaluate the differences in sleep parameters between the groups. For variables with statistical significance, the Student-Newman-Keuls test was performed for pairwise comparisons between the groups. The χ^2^ test was used for rate comparison between the groups. P<0.05 was considered to indicate a statistically significant difference. All analyses were conducted using SPSS 13.0 statistical software (SPSS, Inc., Chicago, IL, USA).

## Results

### Demographic and clinical data of two groups

According to the statistical tests, various data complied with the normal distribution. No significant differences were identified for gender, age, body mass index (BMI), disease course, number of epileptic seizures per month (<3) and blood VPA concentration (all were within the normal range) between patients in the E-AED I, E-AED II and ECO groups. At the night of the recording and in the 72 h prior to the recording, none of the patients presented with clinical seizures, indicating that the data were balanced and comparable (Table I).

### Results of polysomnography

According to the statistical tests, no significant differences were identified in NREM sleep phase 2, NREM sleep phase 3, NREM sleep phase 4, sleep latency and the total sleep time between the three groups (P>0.05). However, significant differences were identified for NREM sleep phase 1, REM sleep, sleep efficiency, the number of times that the patient awoke and the number of REM phases appearing throughout the whole-night sleep between the three groups (P<0.01; Tables II and III).

For indicators with statistical significance, pairwise comparisons between the three groups were performed subsequently.

### Comparisons between the E-AED I and E-AED II groups

In the E-AED II group, NREM sleep phase 1 was significantly prolonged (P<0.05), REM sleep was significantly shortened (P<0.05), sleep efficiency was reduced (P<0.05), the number of times patients awoke during sleep was significantly increased (P<0.05) and the number of REM phases appearing during the whole-night sleep was significantly reduced (P<0.05), when compared with the E-AED I group (Table IV).

### Comparisons between the E-AED II and ECO group

In the E-AED II group, NREM sleep phase 1 was significantly prolonged (P<0.05), REM sleep was significantly shortened (P<0.05), sleep efficiency was reduced (P<0.05), the number of times that the patient awoke was significantly increased (P<0.05) and the number of REM phases appearing during the whole-night sleep was significantly reduced (P<0.05), when compared with the ECO group (Table V).

### Comparisons between the ECO and E-AED I groups

No significant differences were identified in NREM sleep phase 1, REM sleep, sleep efficiency, the number of times that the patient awoke throughout the night and the number of REM phases appearing during the whole-night sleep (P>0.05)between the two groups (Table VI).

## Discussion

Sleep is an important component of daily life and is capable of influencing the occurrence, threshold and duration of epileptic events, while antiepileptic drugs are capable of affecting sleep, waking and the sleep structures of patients. Certain causal associations may exist between antiepileptic drugs and dyssomnia in children with epilepsy (12). Antiepileptic drugs may alter sleep patterns, which appears to be independent of their anticonvulsant effects (13). Sleep structure disorder, daytime drowsiness and difficulty concentrating often appear clinically following the administration of antiepileptic drugs and are on the increase. Numerous epileptic patients suffer from chronic dyssomnia with the resultant consequence being that patient compliance, and that of their parents, for the administration of the antiepileptic drugs reduces. This results in poor control of the epilepsy, which in turn influences the patients’ life, education and the effectiveness of the treatment. Therefore, the sleep problems of epileptic patients severely affect the physical and mental health and the daily life of patients.

Antiepileptic drugs are capable of influencing sleep at night (14). VPA is a drug commonly administered to epileptic patients. A number of studies have investigated the effect of VPA on sleep structures, however, the results of the studies are inconsistent. Although results in the literature vary, the main reason for this lies in the difference of methods used between the studies, including changes in the population under investigation, drug dosages and timing, the duration of treatment, the method for controlling epileptic seizures and the influence of alternative antiepileptic drugs (15). In the present study, none of the epileptic patients presented with dyssomnia problems prior to the administration of VPA and had no epileptic seizures on the night of the recording or in the 72 h prior to the recording, with the number of epileptic seizures per month being <3. Therefore, the effects of epileptic seizures and the sleeping conditions of the patients were excluded. To date, there have been no studies investigating how the length of VPA administration may cause a change in the sleep structures of epileptic patients. The present study observed that the sleep structures are altered in epileptic patients who have been administered VPA for >3 months.

In the current study, comparing the sleep structures between patients in the E-EAD II group with those in the ECO and E-AED I groups demonstrated that for patients in the E-AED II group, NREM sleep phase 1 significantly increased, REM sleep duration significantly reduced, the number of times that the patient awoke increased, sleep efficiency decreased and the number of REM phases appearing during sleep was reduced, while no significant differences were identified in the NREM sleep phases 2, 3 and 4, sleep latency and total sleep time. Between patients in the ECO and E-AED I groups, no significant differences were identified in the sleep structures. Therefore, VPA is capable of damaging the sleep structures of epileptic patients by increasing NREM sleep phase 1, reducing REM sleep, increasing the number of times that the patient awakes during the night, reducing sleep efficiency and reducing the number of REM phases. In accordance with the results from the study by Legros and Bazil (3), Racaru *et al* (12) pointed to the comparison between the VPA group (single-drug treatment or combination treatment) and the group who had not been administered VPA. The REM sleep time of patients in the VPA group was markedly reduced and the time taken to fall asleep again following waking in the VPA group was shorter compared with that of the patients in the group who had not been administered VPA.

Multiple studies investigating the effects of antiepileptic drugs on dyssomnia have indicated that epileptic patients have a greater number of sleep interruptions compared with subjects who do not have epilepsy. Among them, sleep apnea, agrypnia and daytime drowsiness are some of the most common causes of dyssomnia (16). Among the common population, the incidence rate of sleep apnea is at least 3%, while polysomnography analysis of investigated epileptic patients revealed that the incidence rate of sleep apnea may be up to 70%. For epileptic patients, seizures may improve following the diagnosis and treatment of sleep apnea (9). Although Li *et al* (9) did not evaluate the correlation of apnea with epileptic seizures, decreased oxygen saturation may cause brain stimulation or chronic sleep deprivation, aggravating the epileptic seizures. VPA is known to cause weight gain, which in turn is capable of aggravating sleep apnea. The possible mechanisms by which VPA may lead to weight gain in epileptic patients are as follows: i) VPA causes leptin resistance by induction, thus, results in patient weight gain (17); ii) single VPA therapy increases the risk of metabolism syndrome in epileptic patients (18); and iii) VPA may cause weight gain by generating insulin resistance, which increases the serum insulin/glucose levels (19). Weight gain is a major risk factor for cardiovascular and cerebrovascular diseases, as well as sleep apnea. However, whether weight gain is capable of aggravating epileptic seizures or is a factor for the sudden unexplained mortality of epileptic patients remains unclear and requires further study. Reduction in sleep quality and duration are associated with weight gain and obesity (20). The longer the duration of sleep, the lower the risk of obesity, namely the patients have a smaller BMI. It is possible that as sleep duration increases, an increasing number of hormones that burn fat are produced (21). Therefore, we hypothesize that sleep structure disorders in epileptic patients caused by VPA may correlate with VPA-induced weight gain. In the present study, although the statistical tests revealed there were no significant differences in BMI between the patients of the three groups, the BMI of the epileptic patients in whom VPA was administered for >3 months exhibited an increasing trend when compared with the patients in the ECO and E-AED I groups. However, this may be due to the small sample size for the various groups and the short duration of VPA administration. Therefore, it is necessary to perform further intensive research into this area.

In conclusion, VPA is capable of altering sleep structures in epileptic patients. In patients in the E-AED II group, NREM sleep phase 1 was significantly prolonged, REM sleep was significantly shortened, sleep efficiency was decreased, the number of times that the patients awakened during the night increased and the number of REM phases appearing during the whole-night sleep was reduced, when compared with the ECO and E-AED I groups. In addition, these observations began 3 months following the administration of VPA. The possible mechanism by which VPA causes changes to the sleep structures of epileptic patients is through weight gain. Furthermore, the present study provides a theoretical basis for scientific and reasonable applications for antiepileptic drugs in the clinic, including i) the effects of the sleep structure changes are alleviated by controlling the patient’s body weight; ii) the drugs with minor side effect or symptomatic treatments should be applied to improve the patient’s sleep and apnea once the epileptic have dyssomnias which influence the patient’s daily life.

## Figures and Tables

**Table I:** Demographic and clinical data of the three groups.

Variables	ECO group	E-AED I group	E-AED II group	P-value
Males (%)	40	45	40	>0.05
Age (years)	15.15±7.795	12.70±5.536	13.95±5.744	>0.05
BMI (kg/m^2^)	18.67±2.47	18.36±1.84	19.98±5.31	>0.05
Disease course (years)	3.34±3.12	2.35±1.43	2.83±2.22	>0.05
Blood VPA concentration (μg)		55.84±4.60	56.15±4.57	>0.05

Continuous variables are expressed as the mean ± SD. Categorical variables are expressed as rate. BMI, body mass index; E-AED I, duration of VPA administration <3 months; E-AED II, duration of VPA administration >3 months; ECO, without VPA; VPA, sodium valproate.

**Table II:** Comparisons of sleep structures between the three groups.

Group	NREM phase 1 (min)	NREM phase 2 (min)	NREM phase 3 (min)	NREM phase 4 (min)	REM (min)
ECO	23.94±13.27	260.02±30.71	47.00±16.57	73.88±13.31	126.19±35.01
E-AED I	29.50±10.61	260.02±30.71	42.79±6.27	70.48±9.62	116.99±12.90
E-AED II	92.10±48.24	240.96±42.92	51.19±8.75	70.96±24.90	70.82±17.69
P-value	<0.01	>0.05	>0.05	>0.05	<0.01

Data are expressed as the mean ± SD. E-AED I, duration of VPA administration <3 months; E-AED II, duration of VPA administration >3 months; ECO, without VPA; VPA, sodium valproate; NREM, non-rapid eye movement; REM, rapid eye movement.

**Table III:** Comparisons of other sleep parameters between three groups.

Group	Sleep latency (min)	Times the patient awoke (n)	Sleep efficiency TST (min)	TST (min)	REM phases (n)
ECO	19.44±10.36	2.40±1.39	91.96±3.14	531.30±59.71	4.55±0.999
E-AED I	17.19±9.52	2.55±1.42	91.98±2.53	523.99±31.90	4.45±0.999
E-AED II	22.67±16.13	7.25±2.86	89.39±2.55	525.11±48.27	3.60±0.995
P-value	>0.05	<0.01	< 0.01	>0.05	<0.01

Data are expressed as the mean ± SD. TST, total sleep time; E-AED I, duration of VPA administration <3 months; E-AED II, duration of VPA administration >3 months; ECO, without VPA; VPA, sodium valproate; REM, rapid eye movement.

**Table IV:** Comparisons of sleep parameters between the E-AED I and E-AED II groups.

Parameters	E-AED I group	E-AED II group	P-value
NREM phase 1 (min)	29.50±10.61	92.10±48.24	<0.05
REM phase (min)	116.99±12.90	70.82±17.69	<0.05
Sleep efficiency (min)	91.96±3.14	89.39±2.55	<0.05
Times the patient awoke (n)	2.40±1.39	7.25±2.86	<0.05
Periods of REM (n)	4.45±0.999	3.60±0.995	<0.05

Data are expressed as the mean ± SD. E-AED I, duration of VPA administration <3 months; E-AED II, duration of VPA administration >3 months; VPA, sodium valproate; NREM, non-rapid eye movement; REM, rapid eye movement.

**Table V:** Comparisons of sleep parameters between the E-AED II and ECO groups.

Parameters	ECO group	E-AED II group	P-value
NREM phase (min)	23.94±13.27	92.10±48.24	>0.05
REM phase (min)	126.19±35.01	70.82±17.69	>0.05
Sleep efficiency (min)	91.98±2.53	89.39±2.55	>0.05
Times the patient awoke (n)	2.55±1.42	7.25±2.86	>0.05
Periods of REM (n)	4.55±0.999	3.60±0.995	>0.05

Data are expressed as the mean ± SD. E-AED II, duration of VPA administration >3 months; ECO, without VPA; VPA, sodium valproate; NREM, non-rapid eye movement; REM, rapid eye movement.

**Table VI:** Comparisons of sleep parameters between the ECO and E-AED I groups.

Variables	ECO group	E-AED I group	P-value
NREM phase (min)	23.94±13.27	29.50±10.61	>0.05
REM phase (min)	126.19±35.01	116.99±12.90	>0.05
Sleep efficiency (min)	91.96±3.14	91.98±2.53	>0.05
Times the patient awoke (n)	2.40±1.39	2.55±1.42	>0.05
Periods of REM (n)	4.55±0.999	4.45±0.999	>0.05

Data are expressed as the mean ± SD. E-AED I, duration of VPA administration <3 months; ECO, without VPA; VPA, sodium valproate; NREM, non-rapid eye movement; REM, rapid eye movement.
